# Prevalence of Musculoskeletal Disorders in Dental Students and Dentists, and Its Relationship With Extra‐Occupational Factors

**DOI:** 10.1111/eje.70071

**Published:** 2025-11-12

**Authors:** Victor Lloro Boada, M. Cristina Manzanares, José López‐López, Anna Pérez Ventura, M. Laura Giovannoni

**Affiliations:** ^1^ Faculty of Medicine and Health Sciences (Dentistry) & Dentistry Hospital University of Barcelona L'Hospitalet de Llobregat Spain; ^2^ Human Anatomy and Embryology Unit, Department of Pathology and Experimental Therapeutics Universitat de Barcelona L'Hospitalet de Llobregat Barcelona Spain

**Keywords:** dental education, dental ergonomics, dental students, musculoskeletal disorder, sociocultural factors, work posture

## Abstract

**Introduction:**

Dentistry is a demanding profession, in which the oral health team is subjected to conditions, both intra‐occupational to the profession (extreme and repetitive postures and movements during the working day) and extra‐occupational, related to the demands of the profession (lack of time for basic needs such as diet, physical exercise, free time, sleep, social relations given the need for continuing education) that put health at risk. As a consequence, dentists and students have a very high prevalence of musculoskeletal disorders (MSDs).

**Objective:**

To determine the relationship between the MSDs of dental students and dentists and extra‐occupational factors.

**Materials and Method:**

In order to be able to analyze the different study factors, a survey was conducted using validated and accepted questionnaires: Pittsburgh Sleep Quality Index, Maslash Burnout Inventory, Exercise, Nordic Questionnaire, Mediterranean Diet, Musculoskeletal Pathology Nordic Musculoskeletal Symptom Questionnaire, Pemberton Happiness Index and Diaz et al. questionnaire.

**Results:**

A total of 178 participants were enrolled in the study. The sample consisted of 56 second‐year students and 39 fifth‐year students, and 74 dentists. The prevalence of musculoskeletal disorders (MSDs) among second‐year students is 83.93%, while it is 87.18% and 91.46% among fifth‐year students and dentists, respectively. The neck is the most common area of concern in all three groups, followed by the lower back and shoulder. Adherence to the Mediterranean diet was low in all three cohorts, and there was no evidence of burnout, a moderate level of physical activity, but decreasing with age, happiness on average, with mild sleep disturbance (moderate in second‐year medical students).

**Conclusions:**

MSDs in dentistry are a multifactorial consequence. Our study has determined that physical activity is the most relevant extra‐occupational factor in modulating the pathology. However, further research is required to gain a full understanding of the relationship between MSDs and different extra‐occupational factors.

## 1 Introduction

Dentistry is a physically and mentally challenging occupation, with many hazards to health for practitioners. The Oral Health team works in a very small, often dark field and in the presence of biological fluids and sprays. Moreover, they often adopt non‐ergonomic practices. These factors contribute to 97.9% of dentists suffering from a high prevalence of occupational musculoskeletal disorders (MSDs) [1, 2, 3].

The musculoskeletal disorders affect the musculoskeletal system; nerves, tendons, muscles, joints ligaments and bone supporting structures sharing inflammation and degenerative injuries with dental work conditions [4]. Symptoms could include pain, tingling, fatigue and discomfort with signs evident as decreased grip strength or range of motion loss along with sensory input deficits related to motor coordination [5, 6, 7]. These afflictions predominantly involve the arms of dentists, ranging on a continuum from transient and mild symptoms to severe problems associated with loss of function. In addition to reducing the quality of life of those who suffer from them, MSDs negatively affect patient care with inefficient movements, longer treatment times due to pain or discomfort produced by some procedures, and often restricted surgery sites in relation to corporations desired guarantees as well as reduced productivity and increased anxiety. These can result in short or long‐term work stoppages by dentists leading to occupational disability. In larger studies, 68% of dental students experience early MSD symptoms and with their relative inexperience and academic pressures [8, 9].

Given this, we believe that dental students need to be trained adequately with good working habits and risk management measures in order to minimise MSDs [10]. Dental ergonomics, which includes workstation design and techniques used in the workplace as well as dental equipment should be considered an improved safety measure for preventing occupational diseases while reducing stress among workers and providing the best work quality of professionals who will ultimately provide comfort to their patients [11, 12].

The multifactorial causes of MSDs extend beyond workplace conditions to encompass organisational, sociocultural, psychosocial and individual characteristics [13]. Factors such as patient load, the absence of a dental assistant, poor lighting, instrument design, static postures, repetitive movements and individual traits (e.g., poor flexibility, muscle strength, insufficient rest, genetics) contribute to the development of MSDs [14, 15, 16, 17, 18, 19, 20].

Burnout, a related issue, arises from chronic work stress exceeding an individual's coping capacity, manifesting as emotional exhaustion, depersonalization and reduced personal fulfilment. Burnout impacts both dentists and patients, leading to errors, disorganisation, reduced care quality, absenteeism, lower productivity and financial losses. Dental students are also susceptible, risking academic underperformance and career abandonment [21, 22, 23, 24].

It is becoming increasingly clear that lifestyle factors such as diet, physical activity, sleep and happiness play a significant role in the development of MSDs. The Mediterranean diet, which is rich in healthy foods, can help to prevent chronic diseases and potentially impact MSDs [25, 26]. Regular physical activity is beneficial for physical and mental health, and can help to prevent work‐related MSDs and alleviate symptoms [27]. Quality sleep is crucial for physical and mental health, and poor sleep can increase anxiety and the risk of various pathologies, including MSDs [28, 29, 30]. Lastly, workplace happiness correlates with productivity, a better work environment, healthy habits and potentially reduced MSD prevalence [31].

Having observed that sleep, stress, burnout, diet and exercise have been identified in the literature as risk factors for MSDs, we decided to see if their association held true in an occupational population affected by MSDs, such as dentists [32, 33, 34, 35, 36, 37, 38, 39].

### 1.1 Objective

To determine the relationship between the MSDs in a population of second‐ and fifth‐year dental students at the University of Barcelona and practicing dentists, and the extra‐occupational factors.

### 1.2 Design

The research design is non‐experimental, descriptive, prospective and cross‐sectional.

## 2 Materials and Method

### 2.1 Study Population and Participant Selection

A study sample is established that includes dental students and dentists, of both sexes and of various ages. As for the students, they study Dentistry at the University of Barcelona; specifically, from the second to the fifth years. Dentists are professionals selected, validated and finally contacted individually through professional networks (LinkedIn) and master's degree students from the University of Barcelona. Failure to meet the inclusion criteria described above defines the exclusion criteria.

### 2.2 Ethics Committee

The protocol of this study has been approved by the *Committee on Ethics and Research with Medicines and Medical Devices* of the HOUB (CEIm‐HOUB).

### 2.3 Data Collection: Survey

In order to be able to analyse the different study factors, a survey was conducted using validated and accepted questionnaires [31, 40, 41, 42, 43] on each of the variables listed in Table 1.

**TABLE 1:** Questionnaires used according to each variable.

Variables	Questionnaires used
Musculoskeletal pathology	Kuorinka Nordic Musculoskeletal Symptoms Questionnaire
Food	Questionnaire of adherence to the Mediterranean diet SEEDO
Burnout	Maslach Questionnaire Burnout Inventory (MBI)
Physical activity	International Physical Activity Questionnaire (IPAQ)
Are	Pittsburgh Sleep Quality Questionnaire (PSQI)
Ergonomics	Ergonomic Factors Questionnaire, Diaz et al.
Happiness	Pemberton Happiness Index (PHI) Questionnaire

The use of questionnaires in educational settings is beneficial. In addition, these methods can assist in the identification and monitoring of individuals at risk and in the development of educational and preventive strategies [19]. In order to confirm the validity and reliability of data obtained from studies using these instruments on an individual's risk factors for MSDD, we believe the following accompanying questionnaires are relevant based on the literature: Pittsburgh Sleep Quality Index (PSQI) [43], Maslash Burnout Inventory (MBI) [23], Exercise, Nordic Questionnaire(IPAQ) [40], Mediterranean Diet (SEEDO), Musculoskeletal Pathology Nordic Musculoskeletal Symptom Questionnaire (KUORINKA), Pemberton Happiness Index (PHI) [31], Diaz et al. Ergonomic factors that cause the presence of muscle pain in students of dentistry questionnaire [44].

All variables and questionnaires were collected in three digital surveys using Google Forms. Each of them has been assigned a corresponding study group. All three have the same parts, with the difference that the survey for fifth‐year students and of the one for dentists has been reduced by eliminating several repetitive questions on some variables (burnout, sleep and happiness) to facilitate feedback from the participants. This was not necessary for second‐year students, where all the variables have all the questions from the questionnaires used.

To collect the data and access the participants, the link to the survey has been made available to second, fifth and various master's degree students. Dentists verified through LinkedIn Premium, from the Catalonia area, were also contacted via private message.

The survey is completely anonymous, confidential and voluntary. To achieve this, all groups were sent the link on the same day to avoid being identified. In addition, at the beginning of the survey all participants must fill in the items ‘do you agree to participate’ and ‘do you accept to sign in the informed consent’.

### 2.4 Statistical Analysis

The collected data will be entered and analyzed in the Excel package, Microsoft Office 2019 (Microsoft Corporation, Washington, USA, 2013) and kept in a secure online database.

## 3 Results

### 3.1 Study Sample

A total sample of 178 participants was collected, after sending out 300 surveys in total, 100 for each group. 56 s‐year students, 39 fifth‐year students and 83 dentists returned the questionnaire. The sample includes individuals of both sexes and of various ages (from 19 to over 61 years). Specifically, dentists have been interviewed, with a wide range of years of practice (from 1 year of experience up to more than 25 years) as well as a wide range of working hours (from 20 h to more than 40 h per week).

### 3.2 Results on the Prevalence of Musculoskeletal Pathology

Among second‐year students we found 83.93% of students with at least one anatomical region affected by MSD, while among fifth‐year students it increases to 87.18% and to 91.46% when it comes to dentists.

### 3.3 Results on the Prevalence of Musculoskeletal Pathology According to the Affected Body Region

The analysis of data from second‐year dental students shows the following prevalence of MSDs (from highest to lowest %) according to the different regions of the body affected: neck 60.8%, lower back 45.1%, shoulders 35.3%, dorsal area 33.3%, wrists 21.6%, hands 17.6%, forearms 13.7% and arms 9.8%.

**FIGURE 1:**
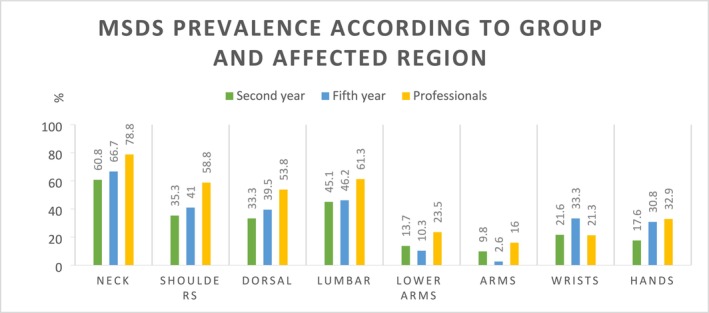
Prevalence of MSDs according to group and affected body region.

When examining the data on fifth‐year students, the following prevalence of MSDs can be observed (from highest to lowest %) according to the different body regions involved: neck 66.7%, lower back 46.2%, shoulders 41%, dorsal area 39.5%, wrists 33.3%, hands 30.8%, forearms 10.3% and arms 2.6%. Finally, studying the information obtained on dentists, we found the following prevalence of MSDs (from highest to lowest %) according to the different parts of the body affected: neck 78.8%, lumbar area 61.3%, shoulders 58.8%, dorsal area 53.8%, hands 32.9%, forearms 23.5%, wrists 21.3% and arms 16% (Figure 1).

### 3.4 Results on Extra‐Occupational Factors Related to Quality of Life (Diet, Burnout, Physical Activity, Sleep and Happiness)

The results obtained on the extra‐occupational factors studied and the quality of life of the three groups demonstrate that, on the one hand, the three groups exhibit comparatively low adherence to the Mediterranean diet. Furthermore, there are no observable differences in the manifestations of burnout or in the level of happiness, or, surprisingly, in the quality of sleep. Conversely, a linear reduction in physical activity is evident from the second level to the professional level. Table 2 provides a comprehensive overview of the findings, including the specific values achieved for each variable and for each group.

**TABLE 2:** Questionnaires results.

	Second year student	Results interpretation	Fifth year student	Results interpretation	Dentist	Results interpretation	Evaluation rules
Diet results	7.98 points	Low adherence (LA) Mediterranean diet (MD)	7.33 points	Low adherence Mediterranean diet	7.58	Low adherence Mediterranean diet	(LA MD): Points < 8 Adherence(MD): 8 > Points
Burnout results							
Emotional exhaustion (EE)	23.77 points (91.42%)	No signs of burnout	11.53 points (57.65%)	No signs of burnout	15.92 points (79.60%)	No signs of burnout	Burnout signs: (EE) > 26; (D) > 9; (PR) < 34
Depersonalization (D)	7.77 points (86.30%)	No signs of burnout	1.2 points (60%)	No signs of burnout	1.77 points (88.50%)	No signs of burnout	
Personal realisation (PR)	36.05 points (106.03%)	No signs of burnout	35.33 points (117.60%)	No signs of burnout	36.45 points (121.50%)	No signs of burnout	
Physical activity results							
Intense activity (I)	1224		1228.80		865.88		LOW: < average AVERAGE: 3 days (I); 5 days (M or I); 5 days ≥ (L + M + I) 600 MET‐min/week HIGH: 3 days ≥ (I or L + M + I) = 1500MET‐min/week; 7 days ≥ (L + M + I) = 3000 MET‐min/week
Moderate activity (M)	419.11		483.60		358.72		
Light activity (L)	473.97		184.10		156.01		
Total activity (T)	2117.09	Average physical activity	1896.50	Average physical activity	1380.62	Average physical activity	
Sleep results	7.81	Moderate difficulty	5.37	Light difficulty	4.73	Light difficulty	0: no sleep difficulty 1–6: Light difficulty 7–13: moderate dif. 14–21: great dif
Happiness results	6.64	Average level of happiness	6.41	Average level of happiness	6.49	Average level of happiness	Very low: 0–3.70 Low: 3.71–5.90 Average: 5.91–7.90 High: 7.91–9.2 Very high: 9.2–10

## 4 Discussion

### 4.1 Analysis of the Prevalence of Musculoskeletal Pathology

As for the prevalence of MSDs, it can be observed that it is increasing in the different groups (83.93% in second year, 87.18% in fifth year and 91.46% in dentists). Likewise, this same pattern of increase is found in the different groups according to the regions affected, where second‐year students have the lowest prevalence, followed by fifth‐year students and, finally, practicing dentists reporting the highest prevalence. This pattern is not seen in the forearms, arms and wrists, as shown in Figure 1.

In all three groups, the most affected region is the neck, followed by the lumbar region, shoulders and dorsal region and, less frequently, the upper limb segments. Considering this pattern of MSDs upsurges as one enters the working world, it can be concluded that these disorders are due to the dental profession, specifically to all the intra‐occupational factors of dentistry practice, analyzed in detail previously, including the non‐ergonomic and static positions, the use of vibrating instruments, repetition of movements and a restricted operating field with poor lighting among others. These results are consistent with the literature, which confirms this relationship [3].

The prevalence of MSDs in dentists and students obtained in this study validates the data extracted from the literature, reaching very similar results—practically identical.

If this prevalence is analysed according to the body region affected in dentists, similar results are also found, especially when compared with those obtained by Shamim et al. [45] However, other authors have reported slightly lower prevalences.

### 4.2 Analysis and Literature on Extra‐Occupational Factors Related to Quality of Life and MSDs

Regarding dietary habits, all subjects evidenced poor compliance with the Mediterranean diet. Contrary to previous studies the present study could not establish a relationship between diet quality and MSD. Earlier research found that a healthier diet lowers systemic inflammation and might relieve symptoms [46].

On the other hand, none of the groups reported excessive levels in burnout factor although stress was present. Also, the rise does not seem to be uniform or lower from the student phase to the professional stage. Therefore, no direct correlation between burnout and MSD was found. Our findings diverge from a study in the general population by Ruela et al. [47], who found stress to be associated with widespread chronic musculoskeletal pain.

The three groups have a similar average physical activity, but the obtained scores show an important difference and hence we cannot say that all of them display the same amount of activity. Dentists have a much lower total score, by 515.88 points compared with the five‐year students and up to 736.47 less than the second years and this reduction in activity is seen not only in the total score, but also across all levels of activities (light, moderate and heavy). We conclude that the physical activity level of the dentists is on average lower compared to students, although it is lower than that of students. For an in‐depth analysis refer to Table 2.

Dentists spend a lot more daylight hours on the job than students do. They also have other concerns that students often do not, like managing the practice and/or staff financially; continuing education both to service patients in daily life, personally with your family or food for workers/washer's household. This means less time in personal activities that nourish well‐being like exercise.

Among our findings here was that those who reported little or no physical activity suffered more MSDs, and in addition to preventing the onset of these conditions, if you already have an MD, regular exercise can make symptoms less severe. Aerobic exercise, for example and stretching are among the physical activities in different types. The former increases tissue oxygen perfusion and hence decreases the intensity of effort required, while in the latter, rest or lower hypertonus [27, 48, 49, 50, 51].

Our results are in line with the literature, that, regular physical activity can help prevent MSDs; moreover, those who suffer from MSDs and exercise have less severe symptoms than those who do not exercise. Among the different types of physical activity, aerobic, exercise and stretching stand out. The former improve the flow of oxygen to the tissues, thus increasing their efficiency, and the latter allow you to relax and reduce muscle tension [27, 48, 51, 52, 53].

In addition to the benefits of exercise for MSDs, it has also been shown a positive association between burnout and exercise [54].

Given the high prevalence of musculoskeletal disorders (91.46%) among dentists, it is crucial to incorporate physical activity and regular stretching into daily routines to mitigate these issues [27, 54].

This is consistent with the probable sleep difficulties observed in second‐year dental students, which were only mild in fifth‐year students and practicing dentists. This difference could be explained by the adjustment problems experienced by second‐year students as they adapt to the demands of the academic and clinical schedules [29]. Because they have become accustomed to the demands of an extended schedule, fifth‐year students, who are holding up well, are subsequently required less at the theoretical level and find themselves working fewer evenings, which hopefully will ensure better sleep. Dentists, on the other hand, allow more time for night‐time rest over other activities, which translates into better sleep quality. Although the role of sleep in MSDs remains undefined, epidemiologic evidence points to an increased long‐term risk of developing MSDs with poor sleep characteristics. This is supported by research showing that the prevalence of chronic pain increases substantially with persistent insomnia [30, 55].

The study surprisingly revealed that second‐year dental students were almost as happy as practicing dentists and only slightly less so than fifth‐year students. The happiness of practicing dentists was similar to that of students, implying the intrinsic rewards of the dental profession as evidenced by practice hours and professional‐personal life management. Furthermore, presumably, the influence of the SARS‐CoV‐2 pandemic on such outcomes is substantial for students and was related to mental health conditions, as well as physical illness; social relationships, including work aspects of family life. The lack of a significant association between MSD and happiness in our study argues for further research to fill a gap in this regard.

The only significant association with MSDs was found in physical activity among all the extra‐occupational factors studied. For this reason, the means to prevent, avoid and alleviate the symptoms of MSDs, dentists/dental students should perform regular physical activity. Still, further research is needed by initiating new trials on MSDs and extra‐occupational factors.

Also, since the literature has shown a significant association between intra‐occupational factors in dentistry and MSDs, in order to prevent MSDs, a series of ergonomic interventions should be implemented in the dental field. Finally, it is important to remember that prevention also includes early detection of symptoms [1, 5, 19, 56, 57].

In summary, a multidisciplinary approach should be implemented to prevent the occurrence of MSDs. It consists of primary prevention, early intervention and ongoing education about the potential impact of dental risk factors.

## 5 Conclusions

A high prevalence of musculoskeletal disorders (MSDs) has been observed among second‐ and fifth‐year dental students at the University of Barcelona and dentists. This prevalence follows an increasing pattern across the different groups, with second‐year students exhibiting the lowest rate and dentists demonstrating the highest rate. In all three groups, the area most frequently affected is the neck, followed by the lower back and then the shoulders. Among the extra‐occupational quality of life factors examined outside the workplace, only physical activity was identified as having a direct correlation with MSDs, both as a predictor and as a mitigating factor. The three groups exhibited comparable levels of physical activity, although dentists demonstrated a markedly lower average. This was observed across all intensities, including light, moderate and heavy. The influence of other lifestyle factors, such as diet, exhaustion, sleep and happiness, on the occurrence and severity of MSDs was inconclusive. However, further research is necessary to elucidate the relationship between MSDs and extra‐occupational factors.

## Ethics Statement

The protocol of this study has been approved by the Committee on Ethics and Research with Medicines and Medical Devices of the HOUB (CEIm‐HOUB).

## Conflicts of Interest

The authors declare no conflicts of interest.

## Data Availability

The data that support the findings of this study are available on request from the corresponding author. The data are not publicly available due to privacy or ethical restrictions.
